# Creatine and cognitive function: Why has physiological plausibility not translated into convincing clinical evidence?

**DOI:** 10.1113/EP094109

**Published:** 2026-10-23

**Authors:** Marco Machado

**Affiliations:** ^1^ Fundação Universitária de Itaperuna Itaperuna Rio de Janeiro Brazil

**Keywords:** blood–brain barrier, brain bioenergetics, cognitive function, creatine, magnetic resonance spectroscopy, phosphocreatine, translational physiology

## Abstract

Creatine is among the most extensively studied and widely used nutritional supplements in sports and exercise science. Over the past two decades, however, interest has expanded well beyond skeletal muscle, driven by growing recognition of the central role played by the creatine–phosphocreatine system in cerebral bioenergetics. Experimental evidence indicates that creatine contributes to neuronal energy homeostasis, mitochondrial function, membrane stability and potentially neuroprotection. This has fuelled growing enthusiasm for the idea that creatine supplementation might enhance cognitive function and benefit individuals with compromised cerebral metabolism. Yet, despite strong physiological plausibility, clinical findings remain inconsistent and often difficult to interpret. In this review, it is argued that the gap between proposed mechanisms and available clinical evidence reflects two distinct challenges: biological constraints on cerebral creatine availability after supplementation, and persistent methodological weaknesses in the clinical literature. We contend that the central challenge for the coming decade is not to identify new mechanisms of creatine action in the brain, but to establish, unequivocally, whether measurable changes in cerebral bioenergetics translate into meaningful cognitive benefit – whether as enhanced performance in healthy individuals or as clinically significant improvement in patient populations.

## Abstract (highlights)

- **What is the topic of this review?**
  This review critically examines the evidence for creatine supplementation as a cognitive enhancer, focusing on why strong physiological plausibility has not yet translated into consistent clinical benefit.
- **What advances does it highlight?**
  The review discusses potential biological constraints limiting cerebral creatine uptake after supplementation (blood–brain barrier transport constraints, transporter regulation, dissociated synthesis by arginine:glycine amidinotransferase or guanidinoacetate methyltransferase), alongside pervasive methodological weaknesses (heterogeneity, multiple‐comparison risk, small samples) underlying the clinical literature, and propose a methodological agenda (including paired ^1^H/^3^ ^1^P‐MRS quantification and pre‐registered trials) for resolving whether creatine's mechanistic promise translates into measurable cognitive benefit.

## 1 INTRODUCTION

Few nutritional supplements rest on as solid a mechanistic foundation as creatine. From early studies of cellular energy metabolism to contemporary models of cerebral bioenergetics, the creatine–phosphocreatine system has long been recognized as a fundamental determinant of ATP availability in tissues with high energy demands. Although research attention has historically centred on skeletal muscle, the brain is among the body's most energy‐intensive organs, consuming approximately 20% of resting energy expenditure to sustain neurotransmission, ionic gradient maintenance and neuronal plasticity (Candow et al., 2023; Forbes et al., 2022; Roschel et al., 2021).

This physiological rationale naturally gave rise to the hypothesis that increasing cerebral creatine availability might enhance cognitive function or mitigate conditions associated with cerebral energy dysfunction. Clinical studies have accordingly investigated the effects of creatine supplementation on memory, attention, processing speed, executive function and a range of other cognitive domains, while a parallel body of literature has begun to explore potential applications in older adults, sleep‐deprived individuals, and patients with traumatic brain injury, neurodegenerative disease and psychiatric disorders (Candow et al., 2023; Forbes et al., 2022; Xu et al., 2024).

This growing enthusiasm for creatine as a putative ‘cognitive enhancer’, however, has not been matched by a corresponding gain in the robustness of the clinical evidence. Although individual studies have reported positive findings, systematic reviews repeatedly highlight substantial inconsistency, methodological heterogeneity and considerable risk of bias (Eckert, 2025; Machado, 2026; Pratt et al., 2026). At the same time, recent advances in our understanding of cerebral creatine physiology raise fundamental questions about the actual magnitude of change achievable through oral supplementation (Candow et al., 2023; McMorris et al., 2024; Pratt et al., 2026).

Here, a critical appraisal of the current state of the field is offered. The aim is not to question the physiological relevance of creatine to the brain – which is regarded as well established – but to examine why biological plausibility has not yet translated into clinical evidence robust enough to support many of the claims currently in circulation. Resolving this question is a necessary step toward establishing whether creatine can fulfil its proposed potential as a scalable, brain‐health‐oriented complement to its well‐established role in skeletal muscle.

## 2 THE PHYSIOLOGICAL RELEVANCE OF CREATINE FOR THE BRAIN IS NOT IN DISPUTE

The importance of creatine for brain function is supported by multiple, independent lines of evidence. The creatine–phosphocreatine system functions as an intracellular energy buffer, stabilizing ATP availability during periods of high metabolic demand. In the brain, this system couples energy production to consumption, thereby supporting neuronal and glial function (Andres et al., 2008; Wallimann et al., 1992; Wyss & Kaddurah‐Daouk, 2000).

Beyond its classical role in high‐energy phosphate transfer, experimental evidence indicates that creatine influences mitochondrial function (Adhihetty & Beal, 2008), oxidative stress control (Lawler et al., 2002; Sestili et al., 2006) and the structural stability of cell membranes (Tokarska‐Schlattner et al., 2012; Wallimann et al., 2011). Animal studies further demonstrate neuroprotective effects across a range of pathological contexts, including hypoxia, cerebral ischaemia and neurodegeneration (Adcock et al., 2002; Prass et al., 2007; Turner et al., 2015; Zhu et al., 2004).

Perhaps the most compelling evidence for the biological importance of creatine to the central nervous system comes from the cerebral creatine deficiency syndromes (Stockler‐Ipsiroglu & van Karnebeek, 2014; van de Kamp et al., 2013). Genetic defects that impair creatine synthesis or transport produce severe neurological deficits, including intellectual disability, behavioural disturbance and epilepsy – findings that unequivocally establish adequate creatine availability as essential for normal brain development and function. The current scientific question, therefore, is not whether creatine matters for the brain, but something quite different: does increasing creatine intake in individuals without a deficiency produce changes in brain availability large enough to generate measurable functional benefit?

## 3 THE PHYSIOLOGICAL BOTTLENECK: INCREASING BRAIN CREATINE IS MORE DIFFICULT THAN INCREASING MUSCLE CREATINE

Much of the success of creatine supplementation in sports science stems from the relatively high capacity of skeletal muscle to accumulate exogenous creatine (Casey et al., 1996; Green et al., 1996; Harris et al., 1992; Liu et al., 2025). In the brain, however, the situation is substantially more complex (Figure 1).

**FIGURE 1:**
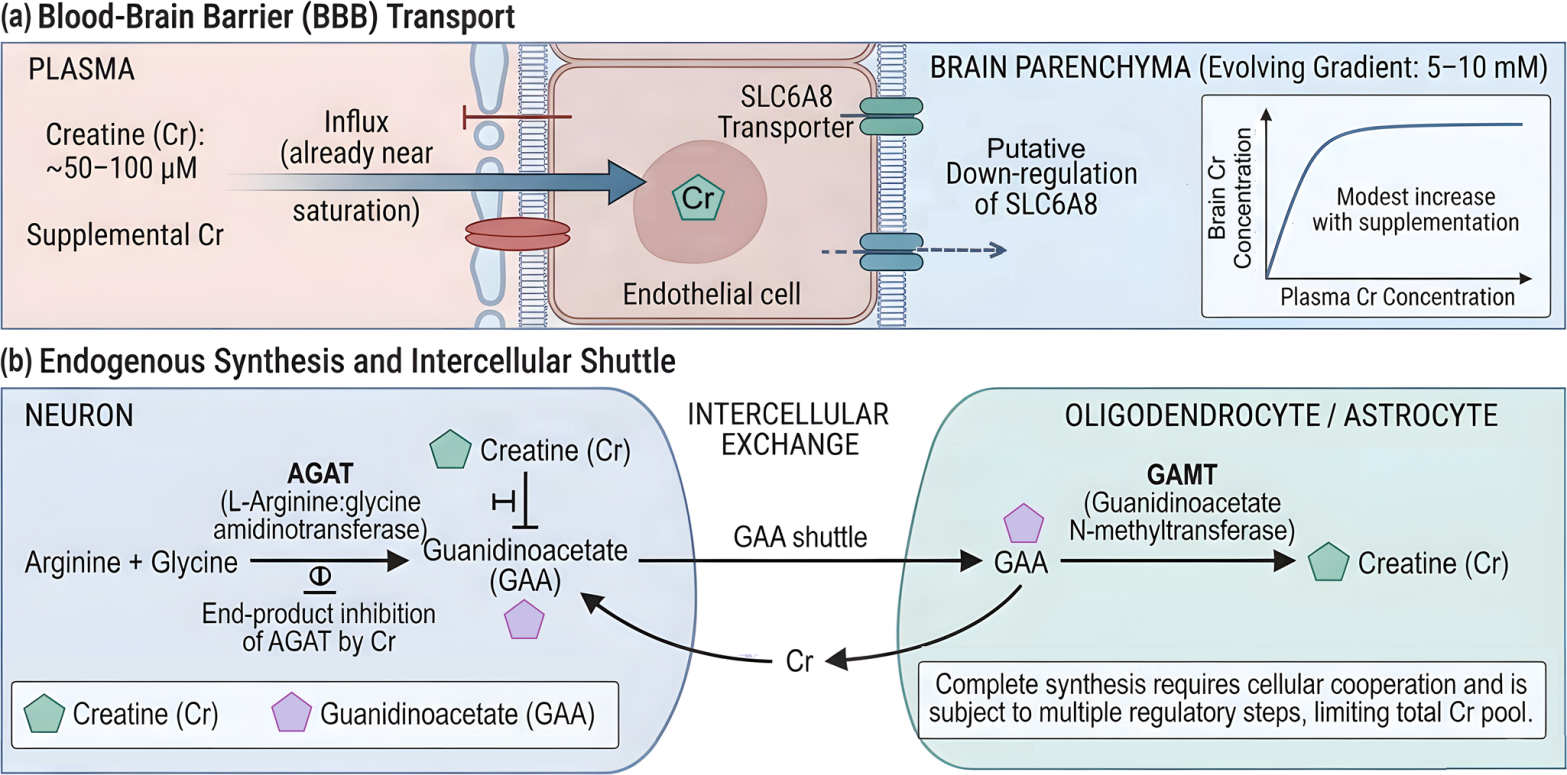
Conceptual schematic illustration of the cerebral creatine bottleneck. Schematic summary of the biological constraints limiting cerebral accumulation of exogenous creatine. (a) Blood–brain barrier (BBB) transport. Plasma creatine (Cr) enters brain endothelial cells via SLC6A8. The large gradient between brain (∼5–10 mM) and plasma (∼50–100 µM) creatine helps explain why supplementation raises cerebral creatine only modestly, though this gradient alone does not establish that the transporter operates near a kinetic ceiling. Chronic substrate exposure down‐regulates SLC6A8 in several cell types; whether this also occurs in BBB endothelium is shown as a proposed mechanism (dashed arrow). (b) Endogenous synthesis and intercellular shuttle. AGAT (neuronal) and GAMT (glial) are rarely co‐expressed, requiring intercellular exchange of guanidinoacetate (GAA) for creatine synthesis; AGAT is further inhibited by creatine itself. Abbreviations: AGAT, arginine:glycine amidinotransferase; BBB, blood–brain barrier; Cr, creatine; GAA, guanidinoacetate; GAMT, guanidinoacetate methyltransferase; SLC6A8, solute carrier family 6 member 8 (creatine transporter).

The blood–brain barrier (BBB) imposes significant restrictions on creatine transport from the systemic circulation (Braissant, 2012). Two distinct claims must be separated here: the empirical observation that cerebral creatine accumulation following oral supplementation is consistently modest, which is well supported (Dechent et al., 1999; Wyss & Kaddurah‐Daouk, 2000), and the mechanistic explanations proposed to account for it, which are considerably less certain. One frequently overlooked aspect concerns regulation of the transporter itself: chronically elevated substrate concentrations have been shown to down‐regulate SLC6A8 in cultured muscle cells (Guerrero‐Ontiveros & Wallimann, 1998; Loike et al., 1988) and in a heterologous expression system (Shojaiefard et al., 2012). To the author's knowledge, this negative‐feedback mechanism has not been directly demonstrated in BBB endothelial cells, and should be regarded as an extrapolation from other tissues rather than an established finding at the BBB itself. If it does operate at the BBB, cerebral creatine transport would be self‐limiting precisely under the conditions in which increased uptake is expected.

A second, separate consideration concerns transporter kinetics rather than transporter regulation. The brain maintains total creatine concentrations on the order of 5–10 mM (Govindaraju et al., 2000; Öngür et al., 2009), far in excess of basal plasma concentrations (∼50–100 µM). This large tissue‐to‐plasma gradient has sometimes been interpreted as evidence that SLC6A8 already operates close to a kinetic ceiling under physiological conditions; however, a large concentration difference does not, by itself, establish proximity to transporter saturation, particularly for an ion‐coupled, secondary active transporter capable of sustaining substantial gradients independently of its fractional substrate occupancy. Structural and biochemical characterization indicates that the human creatine transporter has high substrate affinity, with an apparent *K*_m_ in the low micromolar range (Yuan et al., 2025) – a value consistent with, but not direct evidence of, appreciable transporter occupancy at physiological plasma concentrations, since this estimate derives from a heterologous expression system rather than kinetic measurement at the BBB in vivo. Direct kinetic characterization of SLC6A8 at the human BBB is, to the author's knowledge, currently unavailable, and this remains an important gap for future work. Raising plasma creatine through supplementation therefore appears to alter this gradient only marginally, which may help explain, at least in part, though it does not fully account for, the modest cerebral increases observed empirically (Dechent et al., 1999; Wyss & Kaddurah‐Daouk, 2000).

Unlike skeletal muscle, the brain also possesses its own capacity for creatine synthesis, pointing to homeostatic mechanisms that help maintain relatively stable tissue concentrations of the compound (Braissant et al., 2001; DeFalco & Davies, 1961). Two enzymes mediate this synthesis: arginine:glycine amidinotransferase (AGAT) and guanidinoacetate methyltransferase (GAMT). These display distinct, largely non‐overlapping cellular distributions – AGAT predominates in neurons, whereas GAMT is more strongly expressed in oligodendrocytes and astrocytes – and the two enzymes are rarely co‐expressed within the same cell (Baker et al., 2021; Braissant et al., 2010). Consequently, complete creatine synthesis within brain parenchyma depends on intercellular exchange of the intermediate guanidinoacetate (GAA), imposing an additional layer of regulatory constraint on the local creatine pool (Braissant et al., 2010). More strikingly still, AGAT is subject to end‐product inhibition by creatine itself, such that a rise in exogenous creatine tends to suppress endogenous synthesis through negative feedback (Tropak et al., 2023). The net increase in the cerebral pool may therefore be substantially smaller than would be predicted from exogenous intake alone, and disentangling the exogenous from the endogenous contribution to cerebral creatine is, in practice, all but impossible without isotopic tracers – a tool still rarely applied in human in vivo studies (Pratt et al., 2026; Rae & Bröer, 2015; Solis et al., 2014).

Together, these characteristics raise a question that is frequently overlooked in the literature: even when supplementation substantially raises plasma creatine concentrations, this by no means guarantees an equivalent increase in brain tissue.

The limited data available support this conclusion. Across the handful of human studies that have used MRS to quantify cerebral creatine before and after oral supplementation, reported increases in total brain creatine have generally ranged from approximately 3% to 10%, with considerable variability related to dose, supplementation duration, brain region and acquisition method (Dechent et al., 1999; Moriarty et al., 2023). Within this small literature, findings are also inconsistent: some studies report measurable increases with regimens of approximately 20 g/day for 1–4 weeks, while others report no detectable change using broadly similar protocols (Moriarty et al., 2023). This modest and inconsistent target engagement is itself an important finding, independent of any effect on cognition, and – together with the measurement limitations discussed below – may partly explain why downstream cognitive effects have been equally difficult to demonstrate consistently.

### 3.1 Measuring the pool: What ^1^H‐MRS can and cannot resolve

A further, frequently underappreciated problem concerns measurement itself. Proton magnetic resonance spectroscopy (^1^H‐MRS) – the principal non‐invasive tool for quantifying cerebral creatine in humans – cannot distinguish free creatine from phosphocreatine, as both contribute to the same spectral peak at ∼3.03 ppm (Candow et al., 2023; Öngür et al., 2009). Studies reporting increases in ‘cerebral creatine content’ are therefore, in reality, measuring the combined Cr + PCr pool, without direct insight into the system's true energetic state (Govindaraju et al., 2000; Solis et al., 2014). Phosphorus‐31 spectroscopy (^3^ ^1^P‐MRS) can resolve this distinction, yielding separate estimates of ATP, PCr and inorganic phosphate; however, its intrinsic sensitivity is roughly 15‐fold lower, necessitating much larger acquisition voxels and, consequently, coarser spatial resolution (Svensen et al., 2026). Compounding this problem, the typical coefficient of variation for ^1^H‐MRS has historically been reported in the range of 5–15%, a magnitude comparable to the effects reported after supplementation (∼5–10%), making it genuinely difficult to distinguish true biological change from technical noise. More recent work using optimized acquisition protocols and higher field strengths has achieved substantially better reproducibility, with coefficients of variation for total creatine as low as 1.7–4.4% (Pratt et al., 2025). This is an encouraging development, but it does not resolve the underlying concern for the existing supplementation literature: most published trials to date have not employed these optimized protocols, and the signal‐to‐noise problem described therefore continues to apply retrospectively to much of the current evidence base, even as it becomes increasingly avoidable going forward. This instrumental limitation is far from trivial: it implies that some ‘negative’ studies may simply reflect an inability to detect real change, whereas some ‘positive’ studies may be overestimating true effects (Candow et al., 2026; Zhu et al., 2004).

This has profound implications for how the clinical literature should be interpreted. If an intervention fails to produce consistent change in the physiological variable that is theorized to mediate its effect – cerebral creatine availability – it seems unrealistic to expect robust, reproducible cognitive benefit downstream.

## 4 WHEN METHODOLOGY PREVENTS RELIABLE CONCLUSIONS

Even granting the hypothesis that supplementation can raise cerebral creatine levels under certain conditions, the challenge remains to show that such changes translate into clinically meaningful cognitive benefit. It is precisely here that some of the principal weaknesses of the current literature become apparent (Candow et al., 2026; McMorris et al., 2024).

First, published studies display enormous heterogeneity across virtually every methodological dimension. Dosing regimens vary widely, as do intervention duration, participant characteristics and the experimental contexts under investigation. Trials conducted in healthy young adults are frequently pooled or discussed alongside studies involving older adults, vegetarians, sleep‐deprived individuals and patients with disparate clinical conditions (Candow et al., 2026; McMorris et al., 2024; Xu et al., 2024).

Nor is there consensus on which cognitive domains are most susceptible to creatine's effects, and outcome measures accordingly vary substantially across studies (Marshall et al., 2026; Xu et al., 2024). Short‐term memory, working memory, selective attention, processing speed, executive function and abstract reasoning are frequently grouped together under the generic label ‘cognitive function’, despite representing neurobiologically distinct processes (McMorris et al., 2024). This heterogeneity in outcome measures also complicates efforts to characterize the evidence as uniformly positive or negative: a recent meta‐analysis reported a statistically significant, small‐to‐moderate benefit specifically for memory, even as overall cognitive function and executive function measures showed no significant effect in the same analysis (Xu et al., 2024) – underscoring that domain‐specific signals should not be conflated with, nor dismissed as equivalent to, a genuine absence of effect.

This multiplicity of outcomes creates conditions that are particularly conducive to false‐positive findings. When numerous cognitive tests are administered simultaneously, some statistically significant differences are expected to emerge by chance alone. In the absence of adequate correction for multiple comparisons and of a pre‐specified primary outcome, interpretation becomes considerably more difficult (Citherlet, 2026; Simmons et al., 2011).

A further recurring issue is small sample size. Many published trials enrol only a few dozen participants, limiting statistical power to detect true effects while increasing the likelihood that observed effects are overestimated. This phenomenon is well recognized in the broader methodological literature and contributes to the low reproducibility documented across many fields of biomedical science (Eckert, 2025; Machado, 2026).

## 5 THE CHALLENGE OF EVIDENCE SYNTHESES

Systematic reviews and meta‐analyses should, in principle, reduce the uncertainty inherent in individual studies. In practice, however, the validity of their conclusions depends entirely on the quality of the included studies and the appropriateness of the statistical methods applied (Candow et al., 2026; McMorris et al., 2024).

In the specific case of creatine and cognition, several systematic reviews have reached apparently favourable conclusions, often highlighting benefits for memory or general cognitive performance. More recent critical appraisals, however, suggest that some of these syntheses have been compromised by substantive methodological limitations, including violations of statistical independence and an overly optimistic interpretation of low‐certainty evidence (Eckert, 2025; Eckert & Pascher, 2023).

Perhaps most tellingly, recent independent regulatory assessments have concluded that the available evidence is insufficient to establish a causal relationship between creatine supplementation and improved cognitive function in healthy individuals. This conclusion does not imply that the underlying hypothesis is wrong – only that the evidence has not yet reached the standard required to support strong claims.

A related source of imprecision concerns the benchmark against which evidence is judged. Much of the literature that has been discussed involves healthy young adults, or experimental manipulations such as sleep deprivation and hypoxia, rather than patients with diagnosed clinical conditions. ‘Clinically meaningful cognitive benefit’ is an appropriate standard for trials in patient populations, but it is a poor fit for studies of cognitive performance in healthy individuals, where the more relevant question is whether creatine produces a measurable, reproducible enhancement of performance under specific conditions, rather than whether it reverses or prevents disease. Throughout this review, ‘clinically meaningful benefit’ is used as shorthand for the broader translational goal of the field; it is acknowledged that this framing should, in future work, be applied more precisely and separately to healthy‐population enhancement studies and to clinical‐population intervention studies, which differ in their appropriate evidentiary standards.

## 6 THE HISTORY OF PHYSIOLOGY TEACHES CAUTION

Modern physiology is replete with examples of biologically plausible interventions that failed rigorous clinical testing. The existence of a plausible mechanism does not, in itself, guarantee clinical benefit (Contopoulos‐Ioannidis et al., 2003).

This distinction is nowhere more instructive than in the field of neuroprotection. Over the past several decades, numerous strategies have been proposed on the strength of compelling physiological mechanisms, yet very few have demonstrated consistent efficacy in large‐scale clinical trials (DeGraba & Pettigrew, 2000; O'Collins et al., 2006).

Creatine may yet prove to be one of these translational success stories (Candow et al., 2026). Equally, it may join the long list of biologically plausible interventions that fail to translate into measurable clinical benefit. At present, we simply do not know.

Acknowledging this uncertainty does not weaken the hypothesis – on the contrary, it strengthens the scientific integrity of the field.

## 7 WHERE MIGHT CREATINE HAVE THE GREATEST POTENTIAL?

Despite these limitations, abandoning research into creatine and brain health would be premature.

One particularly promising possibility is that creatine's effects are most evident under conditions of cerebral metabolic stress. Sleep deprivation (Gordji‐Nejad et al., 2024), hypoxia (Turner et al., 2015), prolonged mental fatigue and states of reduced energy availability more broadly (Candow et al., 2026) may represent scenarios in which even modest alterations in cerebral bioenergetics become functionally significant.

By the same logic, populations with impaired cerebral energy metabolism may respond differently to supplementation. Ageing, mild traumatic brain injury, stroke and certain neurodegenerative diseases constitute pathophysiological contexts in which the hypothesis remains biologically plausible and merits rigorous investigation (Li, 2026; Roschel et al., 2021). Within this broad category, future work would benefit from stratifying candidate conditions by the proposed mechanism of benefit – for example, bioenergetic support in states of chronic ATP deficit, buffering of intracellular acidosis, or attenuation of neuroinflammation – rather than treating neurodegenerative disease as a single, mechanistically uniform target. Such stratification could help clarify which specific conditions, and which stages of disease, are most likely to benefit from a bioenergetic intervention of this kind.

There is evidence that vegetarians and vegans, who typically have lower dietary creatine intake and correspondingly lower muscle creatine stores, show a more pronounced cognitive response to supplementation (Benton & Donohoe, 2011). The extension of this argument to cerebral creatine specifically warrants caution: the cross‐sectional ^1^H‐MRS study most directly relevant to this question found comparable total brain creatine concentrations between vegetarians and omnivores despite markedly different dietary intake, a finding more consistent with endogenous synthesis buffering the brain against dietary variation than with a lower cerebral baseline in vegetarians (Solis et al., 2014). Lower skeletal‐muscle creatine in vegetarians should therefore not be assumed to extend to the brain without direct supporting evidence.

Other candidate moderators of the response to supplementation deserve more explicit consideration. Subgroup analyses in a recent meta‐analysis reported more consistent cognitive benefit among older adults, individuals with underlying disease and women (Xu et al., 2024), although the biological basis for these differences remains unclear. Individual variability in response is also well documented in the muscle literature, where a substantial minority of individuals are classified as creatine ‘non‐responders’ on the basis of baseline creatine stores and other factors (Kerksick et al., 2026); whether an analogous responder/non‐responder distinction applies to cerebral creatine and cognition has, to the author's knowledge, not been systematically examined. Individuals carrying reduced‐function polymorphisms in SLC6A8 represent a further, largely unstudied candidate population who may plausibly show a greater ‘physiological headroom’ for response.

Identifying such subpopulations, in which greater ‘physiological headroom’ exists for a measurable response, may be a more productive strategy than pursuing average effects in heterogeneous samples.

Even in these scenarios, however, future studies should look beyond isolated cognitive outcomes. Establishing a coherent causal chain will be essential: supplementation → increased brain creatine → change in physiological biomarkers → clinically relevant functional improvement.

## 8 AN AGENDA FOR THE NEXT DECADE

Advancing the field will require a deliberate shift in priorities. Rather than accumulating further small, heterogeneous studies, the field should prioritize methodologically robust trials capable of answering fundamental physiological questions (Eckert, 2025; Machado, 2026).

Such studies should incorporate protocol pre‐registration, clearly defined primary outcomes, adequately powered sample sizes, rigorous risk‐of‐bias assessment, and, wherever feasible, direct quantification of cerebral creatine using magnetic resonance spectroscopy.

It would also be valuable for future studies to quantify free creatine and phosphocreatine separately via ^3^ ^1^P‐MRS, rather than relying exclusively on the combined peak obtained through ^1^H‐MRS. Only with this distinction can one determine whether supplementation alters not merely the total creatine pool but, specifically, the PCr/Cr ratio – the parameter that reflects the system's buffering capacity and its coupling to mitochondrial creatine kinase. Concurrent monitoring of plasma creatine concentrations alongside urinary creatinine excretion and, where feasible, the use of isotopically labelled creatine would together represent the methodological gold standard for substantially clarifying the question of effective cerebral transport. Pairing plasma creatine measurements with paired ^1^H/^3^ ^1^P‐MRS assessments would additionally allow investigators to test directly whether changes in cerebral Cr or PCr scale proportionally with changes in plasma creatine, helping to distinguish a genuine dose–response relationship from a ceiling effect potentially consistent with transporter saturation, although such measurements alone cannot establish transporter kinetics directly and would need to be interpreted alongside the kinetic limitations discussed above.

## 9 CONCLUSION

Creatine remains one of the most promising tools for investigating human cerebral bioenergetics. Its physiological relevance is beyond dispute, and the proposed mechanisms are biologically plausible. Plausibility, however, should not be mistaken for clinical proof.

The current literature suggests that enthusiasm for creatine's potential cognitive benefits has outpaced the available evidence. Substantial questions remain regarding the magnitude of cerebral creatine increase achievable through supplementation, the physiological contexts in which such increases are functionally relevant, and their ultimate capacity to yield meaningful cognitive benefit, whether understood as performance enhancement in healthy individuals or as clinically significant improvement in patient populations.

The central challenge for the years ahead will not be to identify new molecular mechanisms of creatine action, but to establish convincingly whether these mechanisms translate into functionally relevant effects in humans. Until this question is answered by rigorous, reproducible research, creatine should be regarded not as a proven cognitive enhancer, but as a physiologically compelling hypothesis still awaiting definitive clinical validation.

## AUTHOR CONTRIBUTIONS

Sole author.

## CONFLICT OF INTEREST

None declared.

## FUNDING INFORMATION

None.

## GENERATIVE AI STATEMENT

The author used Claude (Anthropic) as a generative AI writing assistant during the preparation of this manuscript. Its use was limited to: (i) English spelling, grammar and language editing; (ii) checking the completeness and formatting of bibliographic references according to the APA 7th edition style, identifying potentially missing references, and verifying the consistency between in‐text citations and the reference list; and (iii) checking whether the revised schematic diagram presented in Figure 1 addressed the specific points raised by the reviewers. The figure was independently revised by the author. The tool was not used to generate original scientific hypotheses, determine which literature to cite, design the study, interpret the results or draw scientific conclusions. All AI‐assisted output was critically reviewed and independently verified by the author, who takes full responsibility for the accuracy, integrity and originality of the manuscript and for the appropriate use and citation of all references.
